# The Validity of the Danish Version of the Canadian Occupational Performance Measure

**DOI:** 10.1155/2020/1309104

**Published:** 2020-04-22

**Authors:** Anette Enemark Larsen, Sonja Wehberg, Jeanette Reffstrup Christensen

**Affiliations:** ^1^Occupational Therapy, Department of Therapist and Midwifery, The Faculty of Health Sciences, Copenhagen University College, Copenhagen, Denmark; ^2^Research Unit for General Practice, Department of Public Health, University of Southern Denmark, Denmark

## Abstract

**Purpose:**

To establish the construct validity of the Danish version of the Canadian Occupational Performance Measure (COPM).

**Methods:**

A cross-sectional study was performed in two settings, a regional hospital and a rehabilitation centre in a community. Including adult clients with a variety of diagnoses, we assessed construct validity by correlating the COPM to the Occupational Self-Assessment (OSA), the five-item World Health Organization Well-Being Index (WHO-5), and the EuroQol-five domain-five level questionnaire (EQ-5D-5L). Further examination of the comparability of the OSA and the COPM was performed in two ways. First, an interrater agreement of the theoretical correlation of the 21 OSA items and the three areas of the COPM was conducted. Secondly, we examined the compliance between the prioritized occupational performance issues (OPIs) and items of the OSA prioritized for change.

**Results:**

The study included a total sample of 112 participants with more than half of the participants (56%) recruited from the hospital. 109 participants had measurements for both COPM and OSA (44% males) with a mean age of 64.7 years (range 16-96 years). All correlations, between the COPM and the OSA, the WHO-5, and the EQ-5D-5L, were low or negligible (*r* < 0.50). Manual examination confirmed a difference in the constructs of the OSA and the COPM. This was demonstrated by a negligible interrater agreement between the items of the OSA and the areas of the COPM, and differences in the prioritized OPIs and OSA items, even if there were some resemblances, were found.

**Conclusions:**

This study suggests that the construct of the COPM provides data different to those obtained with the standardized measurements included for comparison. The present study supports the assumption that the COPM can detect unique OPIs that clients want to do, need to do, must do, or are not satisfied with the way they do.

## 1. Introduction

The Canadian Occupational Performance Measure (COPM) is designed to help clients identify and prioritize issues in the important occupations they encounter in their lives [1]. These occupational performance issues (OPIs) reflect the clients' issues and wishes with respect to everyday life in three areas: self-care (what a person needs to do including personal care, functional mobility, and community management), productivity (what a person is obliged to do, including paid or unpaid work, household management, and school and play), and leisure (what a person wants to do, including quiet recreation, active recreation, and socialization) [1]. Based on the assumption that occupations are meaningful and help organize time and bring structure to life ([2], p.21), the COPM collects data that reveal which occupations help structure human lives. In the COPM, the clients identify their OPIs of which they prioritize up to five to be the focus of the intervention. Then, they evaluate their performance (COPM-P) and satisfaction with that performance (COPM-S) of their prioritized OPIs [1]. This helps to understand which occupations the clients' value and shed light to how the clients perceive their occupational competencies. Furthermore, the COPM is based on an occupational perspective conceptualized by the Canadian Model of Occupational Performance and Engagement (CMOP-E) [2]. This perspective includes the assumption that a connection exits between being engaged in and performing occupations and the status of humans' health and sense of well-being. Following this in the profession of occupational therapy (OT), occupational performance is believed to influence health and well-being ( [2], p.21).

The most prominent use of the COPM is as an initiated interview that helps clients to determine their OPIs, since this enhances congruence between the clients' perceived needs, wishes and priorities, and the professionals' clinical judgment [3, 4]. This congruence is important as studies have shown that the clients' views may differ from the health professionals' views [4–6]. Furthermore, the COPM documents whether clients perceive improvement in their OPIs, making the COPM a valued measurement in OT interventions [7, 8].

Internationally, the clinimetric properties of the COPM have been widely examined. A review including 19 studies across languages (e.g., Dutch, Norwegian, and Swedish) concluded that the COPM is a valid, reliable (test–retest), responsive, and feasible measurement [9]. Eleven of the 19 studies addressed validity, i.e., the degree to which a measurement measures the construct(s) it purports to measure ([10], p.4), confirming the COPM as a valid measure of occupational performance [9]. Given the lack of a gold standard related to the COPM's construct, most validation studies have examined the convergent validity, i.e., that positive correlations can be obtained between the COPM and measurements with a similar construct ([11], p.172), and divergent validity, i.e., that no or only slight correlations can be obtained between the COPM and other measurements (with a different construct) ([11], p.172). Since the COPM does not address the same items as other measurements, convergent validity was confirmed in former studies with moderate correlations, and divergent validity with low or negligible correlations [12–15], interpreting a correlation coefficient above 0.9 as very high, 0.71 to 0.9 as high, 0.51-0.7 as moderate, 0.31-0.5 as low, and below 0.3 as negligible [16].

The COPM has existed for more than 25 years, and ever since it was translated to Danish in 2000, it has been widely used in Danish healthcare [17, 18]. However, as the former Danish versions were not cross-culturally validated, this study is part of a cross-cultural validation initiated by the Danish association of occupational therapists in 2015 with the release of the 5^th^ version of the COPM. As said by Baker et al., when translating measurements, key constructs of health and semantic meaning may be misinterpreted or misconstrued, leading to invalid results and misplaced interventions [19]. To overcome this, Baker et al. suggest a cross-cultural translation process examining six equivalences [19]. Since the COPM examines a construct, occupational performance, determined by individual and cultural perceptions, especially examining the cultural equivalence of the COPM in Danish seemed important. Thus, in this cross-cultural validation process, we started addressing the first three equivalences (conceptual, item, and semantic) when conducting a new translation of the measurement to Danish [19]. During this process, we discovered some issues regarding the construct and the administering of the measurement [20], which we addressed in the Danish manual with the approval of the authors of COPM. One of these was, that when administering the COPM, the OTs should have their clients address issues of occupational performance, despite that the authors provided examples of tasks in the Appendix A of the 5^th^ version of the COPM manual. In our understanding, determining issues on the level of task might affect the measurement's content validity. To further explore this matter, we have conducted a study focussing on the content validity of the COPM-DK, as can be seen elsewhere in this journal [21]. Another comment was for the OTs to keep their clients from scoring occupations that the clients had not yet performed, as this might affect the measurement reliability. Having completed the translation, we believed to have obtained a cultural equivalent version of the COPM, the COPM-DK, and demonstrated an adequate face validity [20].

In the normal process of a cultural translation procedure, three more steps are described [19]. The first of these is operational equivalence. Based on the need to clarify the administering of the COPM we discovered in the translation process, we decided to conduct a study that examined the clinical utility of the COPM in Danish contexts [6]. The next step is to examine measurement equivalence, i.e., the clinimetric properties of the translated version. Since the COPM's clinimetric properties have been examined worldwide with good results, one could argue that these findings were applicable in a Danish context. However, recalling the findings of issues in our first study that might negatively influence the validity and the reliability of the measurement, we thought it most optimal to continue our cross-cultural translation process with the COPM-DK.

Thus, the present study begins the process of examining the measurement's equivalence [19, 22]. A measurement's equivalence is defined as whether the different versions of the questionnaire have acceptable measurement properties and refers to the comparability of the measurement properties of the instruments in the different language versions ([22], p.119). It examines if the clinimetric properties in terms of reliability, responsiveness, and construct validity are maintained cross-culturally ([19], p.433). This paper describes the examination of the construct validity of the COPM-DK, i.e. the degree to which the scores of the COPM-DK are consistent with hypotheses regarding the relationship with other measurements ([11], p.169), examining the validity by correlating the COPM-DK with the Occupational Self-Assessment (OSA) [23], the EuroQol-five domain-five level questionnaire (EQ-5D-5L) [24], and the five-item World Health Organization Well-Being Index (WHO-5) [25, 26]. The OSA is a self-reported assessment on occupational behavior based on Kielhofner's Model of Human Occupation (MoHO) [27]. We included the OSA in this study, as Stuber and Nelson in an earlier study demonstrated moderate correlation [28]. The EQ-5D is a widely used generic health-related quality of life instrument aiming to describe population health and health outcomes in clinical trials and health economic evaluations [29]. The WHO-5 is a short, generic, and global rating scale measuring subjective well-being and among the most widely used questionnaires assessing subjective psychological well-being [25]. We included the two latter measurements based on the assumption that performing and engaging in occupations' influence but is not the same as health and well-being; thus, we expected some although low correlations.

However, even if the study of Stuber et al. showed moderate correlations and examining the clients' perceptions of their occupational competences and values, i.e., the constructs of the OSA, could be presumed to correlate with the occupational performance issues found with the COPM, we were skeptical of the comparability of the OSA and the COPM. Thus, to substantiate our findings, we included a more in-depth analysis of the correlation of the OSA and the COPM.

This led to the following research questions:

1. To which degree do the clients' ratings of COPM-P and COPM-S correlate with ratings from the OSA, the EQ-5D-5L, and the WHO-5? The examination is based on the following hypotheses:

- To demonstrate the convergent validity of the COPM-DK, we expected the sum scores of the COPM-P and the COPM-S to be moderately correlated to the sum scores of the OSA-C and OSA-V, respectively
- To demonstrate the divergent validity, we expected low correlations between the sum scores of COPM-P and the three active components of the EQ-5D-5L. Likewise, low correlations were expected between the COPM-S and the WHO-5 and the COPM-S and the two affective components of EQ-5D-5L

(2) To which degree are the constructs of the OSA and the COPM comparable? This examination was based on the following two questions:
  - How does the three areas in the COPM and the 21 items of the OSA correlate?
  - Which agreement can be found between the prioritized OPIs of the COPM and the items of the OSA that the clients consider priorities for change?

## 2. Materials and Methods

This study is conducted with the same inclusion methods and populations as described by Enemark Larsen et al.; therefore, the method description partly reproduces their wording [21].

### 2.1. Design, Setting, and Participants

In the study period from February to August 2016, a quantitative single-group pretest design was utilized to capture the relevant data from the four included measurements. The study took place in two settings in the capital region of Denmark. The first setting (A) was a regional hospital, including participants from a hand- and a knee surgery department, i.e., the populations referred to as AH and AK. The second setting (R) was a community-based rehabilitation centre, including participants from in- and out-patient departments, i.e., the populations referred to as RI and RO. The participants were included subsequently after being referred to rehabilitation. The inclusion criteria were age above 18 years and able to speak and understand Danish and to participate in the data collection.

Four OTs from each of the two settings offered to volunteer in the study (eight in total). Two OT research assistants, not employed at the settings, were included to administer the OSA. Thus, together with the first author, a total of 11 OTs participated as assessors in the study.

Together with the first author, another 11 OTs from different regions of Denmark were invited to participate as raters. Eight were experienced OT practitioners (three working in hospitals, two working in the community, and two working on a mental hospital), and three were lecturers from OT educations in universities or university colleges.

### 2.2. Measurements

#### 2.2.1. The COPM-DK

The COPM is a semistructured interview in five steps, in which the OTs helped the participants identify and score their perceived OPIs [1, 30]. In this study, examination of the COPM-DK's validity was based on how the participants scored the prioritized OPIs concerning their *performance* of the OPIs (the COPM-P), and how *satisfied* they were with these performances (the COPM-S). Both scores are VAS scores going from “1” representing the lowest performance or satisfaction to “10” representing the highest performance or satisfaction. The new Danish version of the COPM was used [20, 30].

#### 2.2.2. The OSA

The OSA is a questionnaire that elicits the participants' perceptions of their occupational status [31], as the items of the OSA encompass the three factors that contribute to the overall occupational behavior as described in the MoHO [27]. *Volition* includes values, likes and dislikes, and self-knowledge, including items like “performs activities I like” and “work towards my goals”. *Habituation* includes how human capacity routinely and efficiently contributes to complete daily occupations, including items like “relax and find myself comfortable” and “do what I need to do”. *Performance capacity* includes the skills you need to perform, including items like “physically perform what I need to do” and “taking care of the place where I live” [23, 27]. In the MoHO, *the environment* includes external matters like objects, others, and context. In the initiate development of the OSA, the environment scales showed inadequate separation statistics, and the OT practitioners did not really used it. Therefore, it was removed from the present version of the OSA [32].

In the OSA, the participants evaluated their competences (the OSA-C) through responses to 21 statements, followed by rating the values (the OSA-V) they inflicted on these statements. In rating competencies, the participants were asked to score each statement on a four-point Likert scale ranging from “1” “I have a problem doing this” to “4” “I do this well”. When rating the values, the participants scored each statement on a four-point Likert scale ranging from “1” “this is not so important to me” to “4” “this is the most important to me”. Thus, in the OSA, the participants indicated how well they were doing in a number of aspects of occupational life and how valuable each aspect was for them [33]. The participants looked over their responses and identified four areas they considered their priorities for change (OSA-P). In this study, we used the cross-cultural validated Danish OSA manual [33, 34].

#### 2.2.3. The WHO-5

The WHO-5 is a questionnaire that measures well-being based on five statements: 1 “I have felt cheerful and in good spirits”, 2 “I have felt calm and relaxed”, 3 “I have felt active and vigorous”, 4 “I woke up feeling fresh and rested”, and 5 “My daily life has been filled with things that interest me” [25].

The participants were asked to rate how well each of the five statements applied to them within the last 14 days on a six-point Likert scale from “5” “all of the time” to “0” “none of the time”. Adding the five ratings together, the sum score can range from 0 “absence of well-being” to 25 “maximal well-being”. In this study, we followed the recommendation to multiply the sum scores by four to translate to a percentage scale from 0 absent to 100 maximal [25].

#### 2.2.4. The EQ-5D-5L

The EQ-5D-5L is a questionnaire that measures health with two measures: the EQ-5D-5L descriptive system and the EQ Visual Analogue scale (EQ VAS). The EQ-5D-5L has been developed to improve the instrument's sensitivity and to reduce ceiling effects, as compared to the EQ-5D-3L. Since no Danish norms have been published so far, making it impossible to combine the five items into a total score, we considered each dimension of the descriptive system on its own. Looking at the five dimensions divided in two, the first three: “mobility, self-care, and usual activities”, comprised an activity component, and the last two: “pain/discomfort and anxiety/depression”, comprised an affective component. Permission to use the EQ-5D paper version has been granted with the ID no 32886.

The participants were asked to respond to each dimension within the included five levels with: “1” “no problems”, “2” “slight problems”, “3” “moderate problems”, “4” “severe problems”, and “5” “extreme problems”. This led to an individual EQ-5D health state expressed with a five-digit health profile by combining the levels on each of the five dimensions. The EQ VAS scale gives a quantitative measure of health from 0 to 100 points, as the participants rated their health on a 20 cm VAS scale with the endpoints labelled “the best health you can imagine (100 points)” and “the worst health you can imagine (0 points)” [24, 29].

### 2.3. Procedure

The inclusion procedure was handled by the eight local OT assessors, who recruited the participants consecutively and participated in administering the COPM interviews. Prior to the recruitment of clients, the assessors were informed of the study procedure in a one-day course held by the first author at each setting. This included detailed oral and written information of the study aim and the study procedure, including what the assessors were expected to do, if the clients gave their written consent to participate. The assessors were also instructed how they should administer the COPM to ensure homogeneity and a uniform procedure. Prior to the inclusion of clients, the two OT research assistants were introduced to the OSA and the study in a three-hour course held by the first author. All ten assessors gave their written informed content prior to the start of the study.

In the study period, all newcomer clients being referred to rehabilitation were asked to participate in the study and received verbal and written information about the study. The clients who consented to participate gave shortly after their informed and written consent [35]. Following this, they received an envelope containing the questionnaires EQ-5D-5L [24] and the WHO-5 [25, 26]. If the participants were unable to fill in the questionnaires, the local OT assessors helped them. No later than three working days after consenting, the COPM-DK interview [20, 30] and the OSA interview were administered. The COPM-DK interviews were administered either by the first author or one of eight local assessors. All the OSA interviews were administered by the two research assistants. The COPM-DK and the OSA interviews were performed in random order, so about half of the clients started with the COPM-DK interview and half with the OSA interview. The interviews were performed in Danish at the practice settings. Both the COPM-DK and the OSA were administered verbally according to the measurement's manual and the training courses, and the assessors and the first authors were blinded for other results than the one they produced themselves.

The examination of the comparability of the OSA and the COPM was performed in two tempi. First, alongside with the data collection from the included clients, a theoretical comparison of the two measurements was performed. Together with the first author, the 11 OT raters examined the construct conformity between the COPM-DK and the OSA. All raters were given a sheet with the items of the OSA and asked to fit each item within one of the three areas of the COPM (S, P, and L). These sheets were combined to enable an examination of the interrater agreement.

The second examination was based on the patient data. For this, the first author manually coded the OPIs from the COPM, dividing all OPIs into the three occupational performance areas as defined by the COPM manual and given each OPI a consecutive number. Similarly, all the OSA items prioritized for change by the clients were gathered. This enabled a descriptive comparison of the individual answers from COPM and the OSA to identify a separate top 10 priority list from each measurement based on all the clients' answers.

The study complied with the ethical principles set by the Danish Ministry of Higher Education and Science and the Helsinki declaration [35] and was registered by the Copenhagen University College with id. no 18-025.

### 2.4. Statistical Methods

The COPM-DK's validity was tested by exploring the conceptual conformity with the three standardized measurements, determined through the correlation coefficients as very high, high, moderate, low, or negligible in line with the abovementioned interpretations [16].

First, we calculated characteristics (*n*, mean, sd, median, and quartiles) for all sum scores of the included measurements as described by the manuals. We calculated all sum scores based only on complete cases; i.e., if one or more answers were missing, the participants data were excluded. We further illustrated sum score distributions by histograms.

Then, we calculated the Spearman correlation coefficients and corresponding 95% confidence intervals. In all cases, if a sum score was available, the sum scores of the measurements were used. However, since a Danish norm-based sum score is not yet available of the EQ-5D-5L, we compared the score of each item, with the sum score of the COPM. Thus, the correlations were examined in the following groupings:

(1) The COPM-P sum score was correlated to
  - the OSA-C sum score
  - the EQ-5D-5L VAS-score
  - each of the three active components of the EQ-5D-5L, i.e., 1 “mobility”, 2 “selfcare”, and 3 “usual activities”
(2) The COPM-S sum score was correlated to
  - the OSA-V sum score
  - the WHO-5 percentage score
  - the EQ-5D-5L VAS-score
  - each of the two affective components of the EQ-5D-5L, i.e., 4 “pain” and 5 “anxiety”

Throughout the study, a *p* value < 0.05 is considered significant. Stata version 16 was used for all analyses [36].

To demonstrate the theoretical degree of comparability between the OSA and the COPM, we calculated the interrater agreement of the 12 participating OT raters, using the Fleiss' kappa statistic. The agreement was determined by this classification: <0.20 poor, 0.21-0.40 fair, 0.41-0.60 moderate, 0.61-0.80 good, and 0.81-1.00 very good ([34], p.404). In case of combined ratings, i.e., that the raters could not determine how the item of the OSA could be fitted into one of the three areas of the COPM, self-care (group 1), productivity (group 2), or leisure (group 3), the rating was classified as a group 4 (indecisive).

To examine the degree of agreement found between the prioritized OPIs of the COPM and the items of the OSA that the clients consider priorities for change, we did a descriptive comparison based on the frequencies of the prioritized OPIs/items of each measurement.

## 3. Results

### 3.1. Study Population

141 participants (*n* = 57 males, 40%) were eligible and met the inclusion criteria, of which 112 participants consented. 109 participants had measurements for both the COPM and the OSA (*n* = 48 males, 44%) with a mean age of 64.7 years (range 16-96 years). About half of the participants (*n* = 63, 58%) were recruited from the regional hospital and the rest (*n* = 46, 42%) from the rehabilitation centre. The entire study population is displayed in population groups with gender and age in Table 1.

### 3.2. The Correlation of the COPM, the OSA, the WHO-5, and the EQ-5D-5L

**Figure 1:**
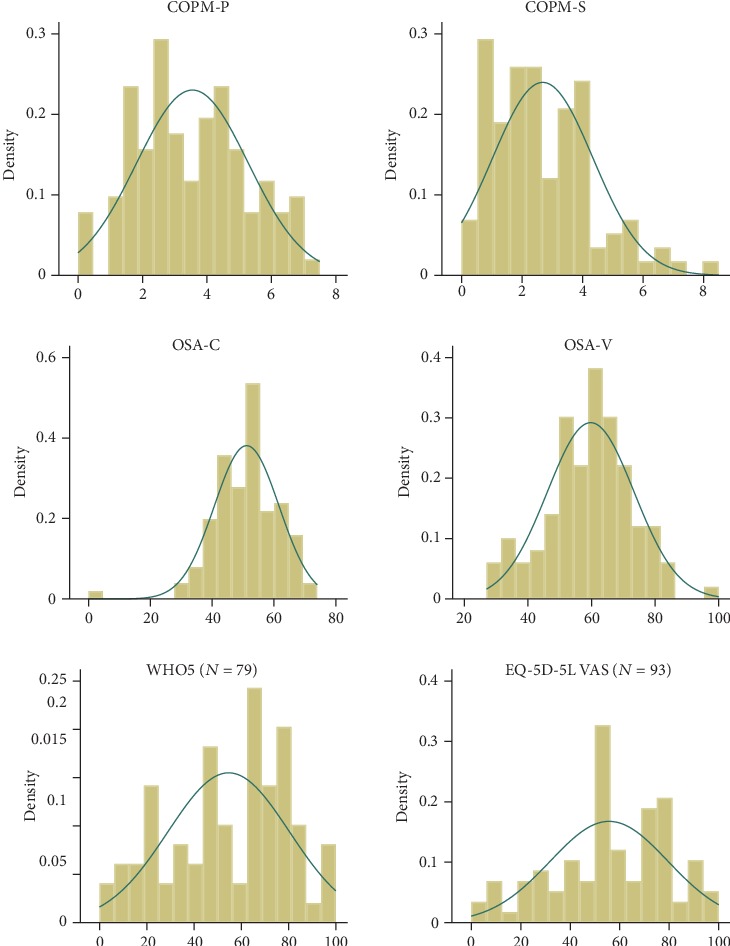
Score distributions COPM-P/S, OSA C/V, WHO-5, and EQ-5D-5L. The figure of the COPM scores shows frequencies of the distribution of the COPM-I values [1–10]. The figures of the OSA and the WHO-5 show the calculated sum scores.

The descriptive scores (mean, SD, range, and median with 25% and 75% quartile) of the COPM, the OSA, the WHO-5, and the EQ-5D-5L can be found in Table 2. The COPM-P score was 3.6 ± 1.7 (mean ± SD) out of 10, and the mean COPM-S score was 2.7 ± 1.7 (mean ± SD). The score distributions are presented in Figure 1.

The correlations between the measurements can be seen in Table 3. The analyses of the total population revealed low or negligible correlations between the COPM and all the other measurements including the OSA scales, with the correlation between the COPM-P and the OSA-C being slightly higher (*r* = 0.20) than the correlation between the COPM-S and the COPM-V, obtaining a negative correlation (*r* = −0.14).

The correlation between the COPM-P/S, the WHO-5, and the split EQ-5D-5L scales was also low or negligible, with the correlation between COPM-P and the third item of the EQ-5D-5L “usual activities” obtaining the highest correlation in the total population (*r* = −0.35) albeit still low (see Table 3).

### 3.3. The Comparability of the OSA and the COPM

The 12 OT raters classified the 21 OSA items into the three areas of the COPM, self-care, productivity, and leisure. On 12 of the 21 OSA items, more than six raters agreed on a classification, as follows: self-care was chosen for three items (25%), productivity four times (33%), leisure two times (17%), and for three of these items, the raters agreed on two of the COPM areas (self-care and leisure), however making them belong to group 4 (the “undecided” group). Following this, the raters only agreed on how nine of the OSA items could be fitted into one of the three COPM areas. The corresponding interrater agreement of the 11 OT raters and the first author was found to be fair (0.29 with 95% confidence interval 0.15-0.43), calculated by the Scott/Fleiss' Kappa statistic [37].

The clients prioritized 495 OPIs in the COPM interviews and 375 items prioritized to change in the OSA interviews. The top ten frequencies of the prioritized OPIs and items to change from the OSA can be seen in Table 4, illustrating differences in the prioritized OPIs and OSA items, even if some resemblances like prioritizing “Shower, taking a bath” (the top priority of the COPM) and “Taking care of myself” (the third most frequent prioritized OSA item) are seen.

## 4. Discussion

### 4.1. Discussion of Results

The aim of the present study was to establish the construct validity of the COPM-DK in relation to the OSA, the WHO-5, and the EQ-5D-5L in clients from various settings in Danish healthcare. Contrary to our first hypothesis, the correlation of the COPM scales with the OSA was low and negligible, whereas our second hypothesis was confirmed since we found low and negligible correlations between the COPM-scales and the WHO-5 and the EQ-5D-5L. Thus, our findings indicate that none of the included measurements' constructs correlate. This finding was further underpinned by the negligible interrater agreement of how the 21 items of the OSA fitted the three areas in the COPM and the illustrated differences in the prioritized OPIs and OSA items. Thus, this study's results support the overall notion, that the COPM provides information that is not obtainable with the predefined items in the three included standardized measures, as they measure an altogether different construct.

#### 4.1.1. The COPM versus the WHO-5 and the EQ-5D-5L

As hypothesized, the correlations of the COPM with the WHO-5 and the EQ-5D-5L measurements were low or negligible, confirming discriminant validity of the COPM. Albeit opposed to the theoretical core assumption of OT and occupational science, in which occupation, health, and well-being are described as being closely related [2, 38], in our hypotheses, we did not expect that this connection would be evident when calculating the correlation coefficient between the measurements. Similarly, in the study of Brekke et al., no correlations between clients' self-reported perceived ability of performing activities and their perceived quality of life were demonstrated [39]. In our study, the highest negative correlation was found between the COPM-S and the third item of the EQ-5D-5L (the activity component) which makes sense as both are regarding performing activities. It is also understandable that there is a little higher correlation between the COPM-P and the perceived mobility, i.e., the second item of the EQ-5D-5L, in clients' who had a knee surgery, whereas the highest correlations were found between the COPM-S versus the EQ-5D-5L anxiety component and versus the OSA-V in the subgroup of old people (aged 64-90) on the inpatient departments of the rehabilitation centre (RI). This can be explained as these clients often are inlaid due to medical issues that challenge their ability to stay in their own houses. Because of their medical condition, discussions of where they should go to live after their rehabilitation are often taken, advising them to move to an old-peoples' home instead of going back to where they lived before. This situation may enforce their anxiety and the importance they place on their satisfaction with and values of their occupational performance and behavior. However, the overall result showed low and negligible correlations as seen in other studies examining the COPM's validity [15, 40, 41]. Thus, the findings underscore the different construct, i.e., (divergent) validity of the COPM-DK when compared to the WHO-5 and EQ-5D-5L.

#### 4.1.2. The COPM versus the OSA

It was surprising that the correlations with the OSA were so low, as Stuber and Nelson found moderate correlations [28]. This difference may be due to the difference in populations, as the group of participants in this study were larger, in average younger, more diverse, and coming from both in- and outpatient settings, whereas the participants in the study of Stuber and Nelson all were inpatients from a hospital. We also used different study procedures, as we included more raters and measurements, and administered both the COPM and the OSA orally. Besides the procedural differences, the negligible correlations do suggest that the two measurements' constructs do not overlap. Indeed, in the OSA, the fixed items are derived from the concepts of volition, habituation, and performance capacity based on the MoHO [27], which are not explicitly included in the COPM. This difference is further underpinned by the negligible interrater agreement in the 12 OTs' attempts to classify the 21 specific items of the OSA into the three occupational areas of the COPM. Albeit some items in the OSA may be considered occupations, e.g., “doing activities I like to do” or “taking care of myself”,, and as seen in Table 4 may resemble some of the most frequently mentioned OPIs, all items in the OSA relate to the competencies that may constitute occupations, with some being solely performance-based like “physically doing what I need to do” or “getting where I need to go”. Thus, the overall construct of the OSA and the COPM does not correlate. The distinction between what data to obtain in the two measurements is important, as performance-based components or body functions are not unambiguously related to occupations or activities [42]. Although minor impairments in body function can have a severe impact on occupational performance, Hansen et al. reported that the recovery of grip strength merely predicted 37% of the performance of daily activities [42], and Ma and Trombly reported that only 38% of an activity could be correlated to body functions [43]. Following this, assessing function cannot predict challenges in occupational performance, and vice versa, and to find out what matters to the clients regarding their occupational performance can only be done by assessing it. Thus, the findings of this study underscore the different construct, i.e., (divergent) validity of the COPM-DK when compared to the OSA, indicating that if you want to know the status of clients' OPIs, the COPM is the best choice.

### 4.2. Discussion of Methods

A limitation in the present study was that we only included clients from the capital region of Denmark. Despite attempts to reach a broad sample, this might limit the generalizability of the results. Possible differences between assessors might also have influenced the results of the COPM and the measures of the OSA. However, as this study did not aim to address intrarater and interrater reliability, we cannot say whether this aspect influenced the results. On the other hand, in clinical practice, we cannot be sure that the same limited sets of OTs will assess all clients with the COPM. Thus, our results may reflect the clinical reality in which to implement the COPM-DK.

We could have exposed some bias to the data collection, when asking the local assessors to help the clients filled in the standardized measurement. However, this was only necessary for five of the clients from the RI population being very confused, and as this inability to participate also made the scoring with the COPM and the OSA impossible, data from these clients are not included in the analysis.

One strength of this study is that it is the first to do a thorough examination of the COPM-DK's validity. Although we used nonparametric statistics, we still treated the sum scores of the COPM-P and the COPM-S scales as continuous data, based on the description from the manual. To enhance the solidity of the study, we included several settings where Danish OTs normally are employed with clients having a variety of issues. Another strength is the carefully selected variety of measurements. In a costly healthcare sector, we should not subject clients to assessments that do not provide necessary data but carefully select measurements that bring unique knowledge. To be sure that the COPM provides data not obtainable from other measurements normally applied within OT interventions, we carefully chose the included measurements as they are frequently used by OTs. Given the clear results with all correlations being low and negligible, we do believe we have documented that the construct validity of the COPM is different from that of the other measurements.

## 5. Conclusion

This study examined the validity of the COPM-DK when correlated with other measurements measuring clients' occupational behavior (the OSA), health (the EQ-5D-5L), and well-being (the WHO-5). All obtained correlations were low and negligible, confirming that the construct of the COPM does not overlap that of the other measurements. Although manual examination revealed some resemblance in the prioritized OPIs and items of the OSA, the low interrater agreement of how the items of the OSA fitted the areas of the COPM underpinned that the constructs of the two measurements are different. In conclusion, the present study supports the assumption that the COPM can detect unique OPIs that clients need, must, or want to do with more satisfaction, being important data in OT practice in Denmark.

## Figures and Tables

**Table 1:** Sample characteristics of the participants (*n* = 109).

	Regional participants (*n* = 63, 58%)		Community participants (*n* = 46, 42%)	
	AK (*n* = 31, 50%)	AH (*n* = 32, 50%)	RI (*n* = 31, 67%)	RO (*n* = 15, 33%)
Gender, m/f	16/15	16/16	10/21	6/9
Age, mean	67	51	76	67
Age, range	Min 49, max 82	Min 17, max 73	Min 64, max 90	Min 31, max 83

AK: regional clients from a hospital, knee replacement; AH: regional clients from a hospital, hand surgery; RI: community clients from a rehabilitation centre, inpatient department; RO: community clients from a rehabilitation centre, outpatient department.

**Table 2:** Scores at admission from all the measurements (COPM, OSA, WHO-5, and EQ-5D-5L) (*n* = 109).

Score at admission	COPM-P	COPM-S	OSA-C	OSA-V	WHO-5	EQ-5D-5L VAS	EQ-5D-5L 1	EQ-5D-5D 2	EQ-5D-5L 3	EQ-5D-5D 4	EQ-5D-5L 5
Range	1-9	1-10	30-74	39-100	8-100	0-100	1-5	1-5	1-5	1-5	1-5
Mean ± SD	3.6 (1.7)	2.7 (1.7)	51.2 (10.5)	59.7 (13.7)	54.7 (25.7)	55.7 (23.8)	2.7 (1.3)	2.1 (1.2)	3.2 (1.2)	2.9 (1.1)	1.7 (1.0)
Median (25-75% q)	3 (2-5)	3 (1-4)	51 (45-58)	61 (51-68)	60 (32-76)	55 (40-73)	3 (1-4)	2 (1-3)	3 (2-4)	3 (2-4)	1 (1-2)

COPM-P/S: COPM performance scale/satisfaction scale; OSA-C/V: OSA competence scale/value scale; WHO-5: the five-item World Health Organization Well-Being Index; EQ-5D-5L: EuroQol-five domain-five level questionnaire; EQ VAS: EQ Visual Analogue scale; q: quartile.

**Table 3:** Spearman correlations for each population group and the total population (*n* = 109).

Population	Correlations COPM-P (*r*_s_)				
	OSA-C (*n*)	EQ-5D-5L Vas (*n*)	EQ-5D-5L 1 (mobility) (*n*)	EQ-5D-5L 2 (self-care) (*n*)	EQ-5D-5L 3 (activities) (*n*)
AH	0.34 (*n* = 32) (-0.01; 0.62)	0.10 (*n* = 24) (-0.32; 0.48)	0.21 (*n* = 26) (-0.19; 0.55)	-0.12 (*n* = 26) (-0.48; 0.28)	-0.39 (*n* = 26) (-0.67; 0.00)
AK	0.11 (*n* = 31) (-0.26; 0.44)	0.32 (*n* = 30) (-0.40; 0.61)	-0.41 (*n* = 31) (-0.67; -0.06)	-0.12 (*n* = 31) (-0.45; 0.25)	-0.25 (*n* = 31) (-0.55;0.12)
RI	0.08 (*n* = 31) (-0.29; 0.42)	0.22 (*n* = 27) (-0.18; 0.55)	-0.29 (*n* = 29) (-0.60;0.08)	-0.31 (*n* = 29) -0.61;0.07)	-0.32 (*n* = 29) (-0.62;0.05)
RO	-0.18 (*n* = 15) (-0.63;0.37)	0.16 (*n* = 12) (-0.46;0.67)	0.02 (*n* = 13) (-0.54;0.57)	-0.24 (*n* = 13) (-0.70; 0.36)	-0.16 (*n* = 13) (-0.65; 0.43)
Total	0.20 (*n* = 109) (0.01; 0.38)	0.27 (*n* = 93) (0.07;0.45)	-0.19 (*n* = 99) (-0.37; 0.01)	-0.24 (*n* = 99) (-0.42; -0.04)	-0.35 (*n* = 99) (-0.51; -0.16)

Population	Correlations COPM-P (*r*_s_)				
	OSA-V (*n*)	WHO-5 (*n*)	EQ-5D-5L Vas (*n*)	EQ-5D-5L 4 (pain) (*n*)	EQ-5D-5L 5 (anxiety) (*n*)
AH	-0.23 (32) (-0.53;0.13)	-0.14 (18) (-0.57; 0.35)	0.26 (24) (-0.1; ,0.60)	-0.27 (26) (-0.60; 0.13)	-0.29 (26) (-0.61; 0.10)
AK	-0.05 (31) (-0.40; 0.31)	0.31 (31) (-0.05; 0.60)	0.31 (30) (-0.05; 0.61)	-0.25 (31) (-0.55; 0.11)	-0.29 (31) (-0.59; 0.07)
RI	-0.05 (31) (-0.40; 0.31)	-0.06 (22) (-0.47; 0.37)	0.01 (27) (-0.37; 0.39)	-0.02 (28) (-0.39; 0.36)	0.00 (29) (-0.37; 0.37)
RO	-0.47 (15) (-0.79;0.05)	-0.04 (8) (-0.72;0.69)	0.27 (12) (-0.36;0.73)	-0.07 (13) (-0.59;0.50)	-0.45 (13) (-0.80;0.13)
Total	-0.14 (109) (-0.32;0.05)	0.20 (79) (-0.02;0.41)	0.26 (93) (0.06;0.44)	-0.16 (98) (-0.35;0.04)	-0.19 (99) (-0.37;0.01)

Correlating the COPM-P and COPM-S sum scores with the sum scores of the OSA-C and OSA-V, the percentage sum score of the WHO-5, the EQ-5D-5L-VAS, and the score of each of the five items from the EQ-5D-5L. COPM-P/S: COPM performance scale/satisfaction scale; OSA-C/V: OSA competence scale/value scale; WHO-5: the five-item World Health Organization Well-Being Index; EQ-5D-5L: EuroQol-five domain-five level questionnaire; EQ VAS: EQ Visual Analogue scale.

**Table 4:** The top ten frequencies of the identified OPIs (COPM) and items prioritized for change (OSA).

COPM/OPIs (*n* = 495)	Frequency (*n*)	Percentage (%)	OSA-prioritized items (*n* = 375)	Frequency (*n*)	Percentage (%)
Shower, taking a bath	37	7.47	Physically doing what I need to do	75	20
Walking (on stairs) up to 2nd floor, to toilet, bedroom	32	6.46	Doing activities I like	60	16
Dressing	24	4.85	Taking care of myself	45	12
Working	23	4.65	Taking care of the place where I live	23	6
Cooking while standing	22	4.44	Getting where I need to go	21	5,6
Biking	16	3.23	Getting done what I need to do	20	5,3
Cleaning	15	3.03	Being involved as a student, worker, volunteer, and/or family member	20	5,3
Eating with cutlery, utensils	15	3.03	Having a satisfying routine	16	4,3
Doing fitness, workout	14	2.83	Accomplishing what I set out to do	16	4,3
Keeping the garden	14	2.83	Taking care of others for whom I am responsible	14	3,7

## Data Availability

Access to data is restricted Complying with European data protection rules, the Copenhagen University College approved the data processing activities regarding this project and registered the project (project number 18-025). We are according to the Danish data protection legislation not allowed to submit the data or give access to the data used for the analyses.
